# Reducing otolaryngology surgical inefficiency via assessment of tray redundancy

**DOI:** 10.1186/s40463-014-0046-2

**Published:** 2014-12-03

**Authors:** Christopher J Chin, Leigh J Sowerby, Ava John-Baptiste, Brian W Rotenberg

**Affiliations:** Department of Otolaryngology- Head & Neck Surgery, Western University, 268 Grosvenor Street, London, ON N6A 4V2 Canada; Departments of Anesthesia & Perioperative Medicine, Epidemiology & Biostatistics, Interfaculty Program in Public Health, Western University, London, Ontario Canada; Center for Medical Evidence, Decision Integrity, Clinical Impact (MEDICI), London, Ontario Canada; Lawson Health Research Institute, London, Ontario Canada

**Keywords:** Otolaryngology, Efficiency analysis, Surgery

## Abstract

**Background:**

Health care costs in Canada continue to rise. As a result of this relentless increase in healthcare spending, ways to increase efficiency and decrease cost are constantly being sought. Surgical treatment is the mainstay of therapy for many conditions in the field of Otolaryngology- Head and Neck Surgery. The evidence suggests that room exists to optimize tray efficiency as a novel means of improving operating room throughput.

**Methods:**

We conducted a review of instruments on surgical trays for 5 commonly performed procedures between July 5th, 2013 and September 20th, 2013 at St Joseph’s Hospital. The Instrument Utilization Rate was calculated; we then designed new ‘optimized’ trays based on which instruments were used at least 20% of the time. We obtained tray building times from Central Processing Department, then calculated an overall mean time per instrument (to pack the freshly washed instruments). We then determined the time that could be saved by using our new optimized trays.

**Results:**

In total, 226 instrument trays were observed (Table 1). The average Instrument Utilization Rate was 27.8% (+/− 13.1). Our optimized trays, on average, reduced tray size by 57%. The average time to pack one instrument was 17.7 seconds.

**Conclusions:**

By selectively reducing our trays, we plan to reduce tray content by an average of 57%. It is important to remember that this number looks at only 5 procedures in the Department of Otolaryngology- Head and Neck Surgery. If this was expanded city-wide to the rest of the departments, the improved efficiency could potentially be quite substantial.

## Background

In 2010 the estimated total expenditure for the Canadian health care system was in excess of $193 billion [1]. This number is projected to grow to $211 billion in 2013 [1], and represents 11.2% of Canada’s gross domestic product (GDP) that year [1]. The value in 1999 was closer to $100 billion, demonstrating there has been a near doubling in total healthcare expenditure over the past 14 years [1]. As a result of this relentless increase in spending, ways to increase efficiency are urgently needed.

Surgical treatment is the mainstay of therapy for many conditions in the field of Otolaryngology- Head and Neck Surgery (OtoHNS), and surgical costs generally exceed those of medical care. Using Endoscopic Sinus Surgery (ESS) as an example, Au et al. reported the novel finding that the average cost of ESS instrument sterilization in the Central Processing Department (CPD) exceeded in a short time the capital costs associated with the surgery [2]. The sterilization process includes cleaning any instruments on the surgical tray that are opened and exposed to the outside environment, regardless of whether or not there was direct patient contact. Often, depending on how the hospital organizes instruments, multiple trays may be used for one procedure. After the instruments are sterilized, they must be re-sorted into their respective trays. Stockert et al. demonstrated that across four different surgical specialties, the amount of instruments used per case was, on average, less than 25% of the instruments contained on a surgical tray (Stockert EW, Langerman A: Assessing the Magnitude and Costs of Instrument Utilization in Otolaryngology Surgical Instrument Trays, unpublished). This suggests that a large proportion of the resources spent sterilizing instruments were for instruments that did not need to be opened. Within this study, OtoHNS fared the worst of assessed specialties – a scant 13% of opened instruments were utilized on average per case.

The evidence suggests that substantial room exists to optimize tray efficiency as a novel means of improving Operating Room efficiency. Systematically assessing the utility of individual instruments could allow for a subsequent reduction in the number of instruments on surgical trays without impacting the surgery or surgeon. This could translate into direct and impactful cost containment in the CPD. Our study objective was to analyze utilization rates for instruments used in five common OtoHNS procedures, hypothesizing that a significant excess of instruments would be demonstrated.

## Methods

**Figure 1:**
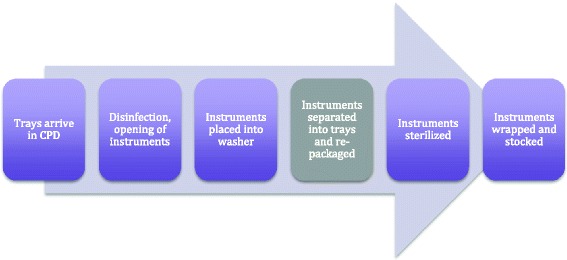
**The sterilization process.** The time for separation and re-packaging of instruments is the most variable.

Many parts of the sterilization process are fixed times, regardless of the number of instruments to be processed. The most variable part, which is when the instrument trays are rebuilt, was the focus of this study. The time to build a tray is recorded to allocate the proportion of the CPD cost to each surgical department. It is this part of the cleaning process that is most time-consuming, most variable and most sensitive to the number of instruments on the instrument tray. At London Health Sciences Centre (LHSC) and St. Joseph’s Healthcare (SJHC) in London Ontario, instruments arrive in CPD and are first quickly rinsed and opened, to allow a more thorough cleaning and removal of gross debris. This stage is known as decontamination and the time is, in most cases, negligible. The trays are then placed into a large washer for a fixed amount of time, regardless of how many instruments are present. Next, the instruments are removed and the trays are rebuilt. If multiple trays are used together for a procedure these instruments are separated and re-packaged into their distinct trays. Lastly, the trays are placed in a sterilizer for a fixed amount of time, and then wrapped and stocked in their appropriate locations. The standard CPD flow is shown in Figure 1.

A review of instruments on surgical trays for 5 commonly performed procedures was performed. Surgical cases by four academic Otolaryngologists at SJHC were examined between July 2013 and September 2013. At the conclusion of the surgery, a sheet listing all available instruments per tray was used to identify which opened instruments were actually used in the case. For some cases, multiple trays were opened. This data was collected and organized into a database using Microsoft Excel 2012 (Microsoft Corporation, Redmond WA). The Instrument Utilization Rate (IUR) was then calculated using the formula:

$$ \begin{array}{l}\mathrm{Instrument}\ \mathrm{Utilization}\ \mathrm{Rate}=\left({\mathrm{I}}_{\mathrm{Used}}/{\mathrm{I}}_{\mathrm{Total}}\right)*100\hfill \\ {}\mathrm{Where}:{\mathrm{I}}_{\mathrm{Used}}=\#\mathrm{of}\ \mathrm{in}\mathrm{struments}\ \mathrm{used}\ \mathrm{in}\ \mathrm{case}\hfill \\ {}{\mathrm{I}}_{\mathrm{Total}}=\#\mathrm{of}\ \mathrm{in}\mathrm{struments}\ \mathrm{in}\ \mathrm{the}\ \mathrm{tray}\hfill \end{array} $$

The total IUR for all identical cases was then calculated. If an instrument was used at least one out of every five times (20% of the time) the tray was opened, it was felt this would merit inclusion on a new optimized tray for that particular procedure. Once these lists were completed, each of the Otolaryngologists was asked for feedback to suggest any instrument additions to ensure that all surgeons would be content with the new surgical trays. No subtractions were made to the trays, ensuring that the trays were not artificially shrunk (which would increase the percent reduction). Focusing on five procedures (septoplasty, septorhinoplasty, skin cancer excision, ESS, and tonsillectomy), new, “optimized” trays comprised of far fewer, but more frequently utilized, instruments were subsequently created.

## Results

In total, 226 instrument trays were observed (Table 1). The average IUR was 27.8% (+/− 13.1). The trays with the highest IUR were the frontal sinus and the sphenoid tray, which both had utilization rates of 50% or more (Table 1). These two trays are not routinely opened, and are only available upon request by the operating surgeon.

**Table 1:** **Tray utilization**

**Tray**	**Number of observed uses**	**Number of instruments**	**Mean utilization (%)**
Skin	3	43	37.21
Adenotonsillectomy	6	34	29.41
Nasal Packing	67	14	21.28
Septoplasty	67	70	20.14
Septorhinoplasty	16	58	39.98
Sinus	45	16	36.39
Image Guidance	11	17	32.09
Frontal	10	6	51.67
Sphenoid	1	2	50.00

The tray that had the lowest IUR was the Septoplasty tray (20.43%). Interestingly, at SJHC, the Septoplasty tray is opened for all ESS cases, septoplasties, and septorhinoplasties, indicating that it may have instruments that are useful for each of these cases, but not necessarily all three of them.

By using a 20% cut-off, the most commonly used instruments were used to design smaller trays and to reduce multiple trays being used routinely. The number of instruments in the old trays, the new trays, and their reductions are listed in Table 2. The average reduction in instruments was 57% across all trays. The ‘Nasal Packing’ tray was incorporated into each of the rhinology trays (Septoplasty, Steptorhinoplasty and Sinus), such that one tray would be opened instead of two or three. The Image Guidance, Frontal, and Sphenoid trays were not reduced and would be used on an as-requested basis, as they were previously.

**Table 2:** **Reduction in tray sizes**

**Tray**	**Number of instruments (Old)**	**Number of instruments (New)**	**Reduction (%)**
Skin	43	27	−37.2
Adenotonsillectomy	34	13	−61.8
Septoplasty	84	33	−60.7
Septorhinoplasty	142	60	−57.8
Sinus	100	36	−64.0

The time to build OtoHNS surgical trays was collected. The data represented 173 trays, with a total of 9445 builds, and reported the average assembly time per tray. Trays with a minimum of 10 instruments, and a minimum of 10 builds, were selected as being sufficiently representative of the time relevant to building the trays of interest. Of a total of 173 trays, 39 met the inclusion criteria, representing 4,541 builds. The mean time per sterilization time per instrument was 17.7 seconds, ranging from 7.6 to 31.6 seconds (2.5 and 97.5 percentiles, respectively).

## Discussion

As the cost of the Canadian healthcare system continues to balloon, new and innovative ways to improve operating room efficiency are continually being sought. Anecdotally, it was noted that many instruments were present but not being used on surgical trays that were opened routinely, and indeed after analysis over 70% of our instruments met criteria for being superfluous to the majority of procedures. It is important to remember that this number looks at only a small number of procedures within the relatively small specialty of Otolaryngology- Head and Neck Surgery. Our novel data strongly suggests that if this type of analysis were to be expanded to a wider array of procedures done by our specialty, to say nothing of other surgical specialties who undoubtedly have the same high level of instrument redundancy, efficiency improvement to the hospital would be substantial.

A question that remains after the trays are reduced is what to do with the removed instruments. Some are completely redundant, while others are potentially needed rarely. One option would be to individually wrap each instrument, and open it only when the specific instrument is requested. A second option would be to wrap the instruments in “groups”, similar to the frontal sinus instruments. In these cases, if the surgeon feels that a particular instrument or set of instruments would be warranted, they could request them and the instruments would then be opened. Given that we based our tray reductions on a “20% usage rule”, as previously described, we believe that these instruments would be infrequently opened and that this would be a reasonable solution that has worked very well for both the sphenoid and frontal instruments at our site (usage rates of 50 and 51.67% respectively). Importantly, once implemented, instrument utilization would be monitored and any instrument that is more frequently requested can simply be added back onto the surgical tray.

This study has several limitations. The cost estimates are relevant in economic terms. Tray building time measurements were obtained from London-based hospitals and may be different at other sites in Ontario or Canada; hence the results may not be seamlessly generalizable, although the basic concept of the study likely is. Potential time-savings during the decontamination process were not incorporated due to a lack of data; however, decontamination times are unmeasured because decontamination occurs in batches and is not considered a significant cost driver in the CPD department.

There are several future directions to study. Other efficiencies could also be anticipated such as shorter set-up times for nurses between cases and smoother flow within surgical cases (as nurses will have an easier time locating the requested instrument). There would also be a significant reduction in the cost of purchasing the surgical instruments to create new trays or replace existing instruments. Both of these concepts pose questions that can be examined with future research in this area. Furthermore, a formal cost analysis should be performed both with our data and that of other services. From an environmental perspective, much has been written about the amount of waste produced from the operating theatre [3,4]. The simple act of shrinking the contents of trays, and combining multiple trays into fewer trays, would immediately reduce the waste produced from the tray wrappings. As well, smaller, more efficient trays should allow for savings in energy, water, and reagent when running the washer. These are all unexplored areas of efficiency improvement that while small individually would over time produce incremental and measurable financial improvements in the Operating Room, and remain as areas for further study. Lastly, there is also the potential for intangible benefits that come with the anticipated faster and simpler set-up in the OR. These benefits include reduced stress in the OR, greater time for surgical learning, and improved flow of surgical cases, particularly when searching for an instrument in high-stress situations. These are all areas of future investigation.

## Conclusion

As the cost of providing healthcare increases, there is mounting pressure to increase efficiency in the operating room. Our novel data has shown that a measurable improvement in efficiency can be realized simply by assessing instrument utilization rates on surgical trays. This technique can be applied both across Otolaryngology – Head & Neck surgery as well as other specialties.
